# Geometric nonlinear diffusion filter and its application to X-ray imaging

**DOI:** 10.1186/1475-925X-10-47

**Published:** 2011-06-05

**Authors:** Eric Michel-González, Min Hyoung Cho, Soo Yeol Lee

**Affiliations:** 1 Department of Biomedical Engineering, Kyung Hee University, 1 Seocheon, Yongin, Gyeonggi, 446-701, Korea

## Abstract

**Background:**

Denoising with edge preservation is very important in digital x-ray imaging since it may allow us to reduce x-ray dose in human subjects without noticeable degradation of the image quality. In denoising filter design for x-ray imaging, edge preservation as well as noise reduction is of great concern not to lose detailed spatial information for accurate diagnosis. In addition to this, fast computation is also important since digital x-ray images are mostly comprised of large sized matrices.

**Methods:**

We have developed a new denoising filter based on the nonlinear diffusion filter model. Rather than employing four directional gradients around the pixel of interest, we use geometric parameters derived from the local pixel intensity distribution in calculating the diffusion coefficients in the horizontal and vertical directions. We have tested the filter performance, including edge preservation and noise reduction, using low dose digital radiography and micro-CT images.

**Results:**

The proposed denoising filter shows performance similar to those of nonlinear anisotropic diffusion filters (ADFs), one Perona-Malik ADF and the other Weickert's ADF in terms of edge preservation and noise reduction. However, the computation time has been greatly reduced.

**Conclusions:**

We expect the proposed denoising filter can be greatly used for fast noise reduction particularly in low-dose x-ray imaging.

## Background

Image denoising is very important in digital x-ray imaging since it may allow us to reduce x-ray dose in human subjects without noticeable degradation of the image quality. Owing to the advent of 2D flat-panel x-ray detector, digital x-ray imaging modalities like digital radiography (DR) or 3D cone-beam CT are now widely used for clinical imaging [1,2]. Taking x-ray images with low x-ray dose is always desired due to the hazardous biological effects of x-rays. However, low dose x-ray images may suffer from high level noises stemming from randomness of x-ray photon fluence and detector noise. There have been many developments of denoising filters that can be used to improve signal-to-noise ratio (SNR) of x-ray images without compromising the image resolution to a noticeable extent [3-5]. In denoising x-ray images, it is critical not to lose detailed spatial information in the original images which is crucial for accurate diagnosis. Recent developments of non-linear iterative denoising filters make it possible to remove noise from the x-ray images while maintaining edge features, which is impossible with linear filtering techniques. In implementing non-linear iterative denoising filters in x-ray imaging, fast computation time is one of the critical factors since x-ray images usually have large sized image matrix. DR images have big matrix sizes as large as 3500 × 4300 for chest imaging and 3600 × 4800 for breast imaging. 3D cone-beam CTs based on a flat-panel detector often make images with the matrix size as large as 1024 × 1024 × 512. For x-ray images of this big matrix size, fast computation is essential for practical use of the iterative denoising filters.

Anisotropic diffusion filter (ADF) is one of the most widely used denoising techniques in medical imaging [6-9]. ADF is believed to have good performance in denoising while well preserving edge features, and it is also often used for edge detection [10-13]. Explicit schemes of ADF needs large amount of computation [14], mostly coming from calculation of diffusion coefficients and gradients at every pixel. To improve the performance of ADF particularly in terms of computation time, we propose an iterative denoising filter considering the local image topology in the nonlinear diffusion model. We have tested the filter performance using synthetically and experimentally obtained x-ray images. We present the performance of the proposed filter in comparison with the Perona-Malik ADF and Weickert ADF with experimental results obtained from low dose 3D micro-CT images and 2D DR images.

## Methods

### A. Modified nonlinear diffusion filter

Perona and Malik proposed the following partial-differential-equation (PDE) based denoising filter on a continuous domain [10]:(1)

where *c*(·) is the diffusion coefficient function based on the image gradient ∇*I *and *I_o _*is the initial image. They suggested two diffusion coefficient functions of the form:(2)

and(3)

where *k *is the noise threshold that controls the amount of diffusion to be applied in the gradient direction. Optimal choice of *k *is known to be very important for successful denoising with the ADF and many kinds of *k *estimators have been proposed for practical applications [15,16].

An explicit discrete form of the Perona-Malik ADF is given by [10]:(4)

where  is the discrete image at time *t, s *and *p *denotes the pixel position in a discrete 2D grid, *Δt *is the time step size in PDE,  is the spatial neighborhood of pixel *s *,  is the number of pixels in the window, and . By implementing Eq. (4), calculation of the diffusion coefficient is most computationally intensive. In most discrete implementations of ADF, the number of neighborhood pixels is often chosen to be four, that is, east, west, north, and south pixels. Therefore, we need to calculate the diffusion coefficients at least four times at every pixel.

One of the limitations of this explicit discretization scheme is the stability requirement that limits the step size *Δt *to less than 1/2*d*, where *d *is the number of directions along which the gradients are calculated [14]. Therefore it takes longer computation time if we choose more number of directions. Despite success of the Perona-Malik model for practical use, it is known to be an ill-posed problem [10,11,17]. To overcome this problem, incorporating regularization into the PDE has been proposed [13,18]. In the regularized methods, the diffusion coefficients are derived from the regularized image rather than from the noisy image to avoid excessive noise effects on the gradient calculation. Other approaches, that modify the diffusion coefficients to take account of local image statistics, have been also proposed. Black and Saphiro have proposed a robust statistical estimation approach in which they used diffusion coefficients derived from the Tukey's biweight function, and they have shown better edge preservation after denoising [16]. You *et al. *explored a variety of anisotropic diffusion equations and they reported some improved results [17]. In all the aforementioned methods, the image features have been simply classified as ∇*I *<*k *for noise regions and ∇*I *<*k *for edge regions and its performance is well known to be successful. On the other hand, Weickert proposed a more strict anisotropic diffusion model where a diffusion tensor is used [11]. The anisotropic model mitigates the limitation of the Perona-Malik model in which the diffusion process stops at edges leaving the noises near the edges. It is believed that the Weickert model outperforms other isotropic models but it takes more extensive computation time.

In this work, we concern about the computation time of ADF in denoising medical images. We have noticed that using only a global *k *in defining the diffusivity function works well in most cases, but more local image information must be incorporated into the diffusivity function for faster convergence. Therefore, instead of using only a global *k *and image gradient in the diffusivity function, we are to use the global noise behavior and the local topology of the pixel intensities in defining new diffusivity functions.

**Figure 1:**
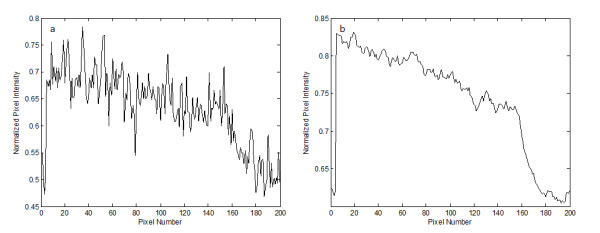
**An x-ray image profile**. (a) A typical profile of a DR image taken with low x-ray exposure level. (b) The profile at the same row of the DR image taken with high x-ray exposure level.

**Figure 2:**
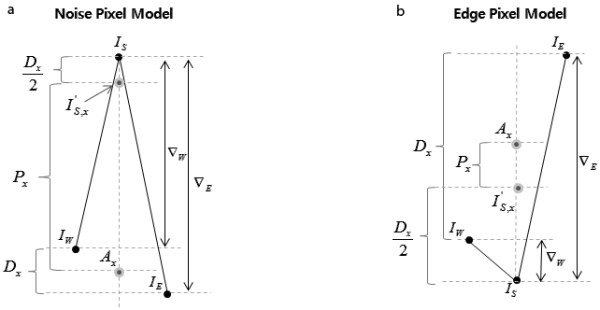
**Pixel classifying models**. (a) A typical noise pixel and (b) a typical edge pixel in the horizontal direction. *s *is the pixel of interest and *E *and *W *are east-side and west-side pixels, respectively.

Figure 1a shows a typical profile along a row in a DR image of an adult rat taken with low x-ray exposure level (40kVp, 67 μA). Figure 1b is the profile at the same row of the DR image taken with higher x-ray exposure level (40kVp, 500 μA). From visual observation of Figure 1, we categorize two conspicuous types of pixels, the noise pixel and the edge pixel. A noise pixel stands for a pixel that has much higher or lower intensity than adjacent pixels having similar intensities. The edge pixel stands for a pixel that is either on an inclining slope or on a declining slope as shown in Figure 2. From the topology of the noise and edge pixels, we define the following parameters:(5)(6)(7)(8)

where *I_E _*and *I_W _*are the image intensities at the east and west neighboring pixels, respectively, and *I_S _*is the image intensity of the pixel of interest. *D_x _*represents the *x*-directional intensity difference in a 3 × 3 window. δ ∈ [0, σ], where *σ *is the standard deviation of the image noise, is an auxiliary parameter to prevent small noise regions from being identified as edges by setting *D_x _*to 0 when |*I_E _*- *I_W_*| ≤ δ. Introducing *δ *to *D_x _*has the effect of combining both global and local information on the diffusion coefficient. For automatic computation of *δ*, we use the median absolute deviation (MAD) as suggested in [16] for calculating *k *in ADFs and used by [6,8]. The average of the neighboring pixels in the *x*-direction, *A_x_*, represents a local intensity reference for a given pixel. From Figure 2, we observe that *P_x _*is greater than *D_x _*at the noise-like pixel and vice versa at the edge-like pixel. We can define parameters *D_y_, A_y_*, , and *P_y_*in the vertical direction in a similar way by considering the north and south pixels. At noise-like pixels, *P_x _*will have a higher value as compared to *D_x _*and we are to define a diffusivity function that allows fast diffusion around the noise-like pixels. Here, we define diffusivity functions that are monotonically decreasing along the *D_x _*or *D_y _*direction and monotonically increasing in the *P_x _*or *P_y _*direction as below:(9)

**Figure 3:**
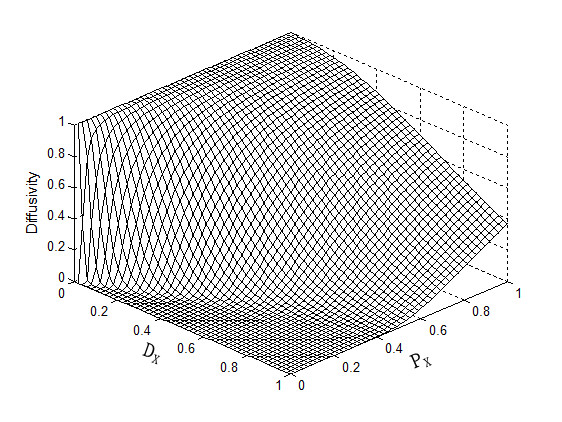
**Diffusivity function**. A perspective view of the proposed diffusion coefficient function when *δ *= 0.

At any noise pixels where *P_x_>> D_x_, c *becomes close to 1 allowing big diffusion. But, at the edge pixels where *P_x_<< D_x_, c *becomes close to 0 allowing small diffusion. As compared to the Perona-Malik diffusion coefficient which is determined by the local gradient magnitude and a global parameter *k*, the proposed diffusion coefficient is determined by the two local parameters, which results in more localized diffusion process than the original ADF. We define only one diffusion coefficient in one direction here, either horizontal or vertical, while Perona-Malik method defines two diffusion coefficients in one direction. Therefore, we can expect computation time saving by factor of two. One example of c(*D_x_, P_x_*) is shown in Figure 3. The discrete implementation of the proposed filter is:(10)

where ∇*_p _*= *I_p _*- *I_S _*(*p *= *E, W, N*, and *S*) represents the difference between the central pixel and one of the east, west, north, and south pixels respectively. Since we use the same explicit scheme used by Perona and Malik for our numerical PDE solver, the stability will depend only on the step size Δ*t*. Previous works [10,17] have pointed that the solution always converges to a steady state under a small step size (normally).

### B. Metrics for filter performance evaluation

For quantitative evaluation of the filter performances in terms of noise reduction and edge preservation, we use the following metrics.

#### Normalized Mean Square Error (*NMSE*)

We calculate *NMSE *of the original and filtered images with respect to the true images when the true images are available. We assume the images taken with much higher x-ray exposure to be the true images since they have much less noise as compared to the ones taken with low x-ray exposure. *NMSE *would represent how the filtered image resembles the true image. *NMSE *is defined by:(11)

where *I_f _*and *I_n _*are the filtered and original images, respectively, and *I_T _*is the true image.

#### Noise Energy Reduction Ratio (*NERR*)

Assuming the noise is white all over the spatial frequency band and the signal is mostly contained in the low spatial frequency band, we take Fourier transform of the original image and the filtered image to transform them to the spatial frequency domain. Then, we apply 2D radial high-pass filter with the cutoff frequency *k*_c_, that is, , to the transformed images to remove the signal components residing in the low frequency band. After the high-pass filtering, *NERR *is then calculated by:(12)

where *u *and *v *are the spatial frequencies in the *x*- and *y*-directions, *D_n _*is the Fourier transform of the difference of the original image from the true (high x-ray dose) image after the high-pass filtering. *D_f _*is the Fourier transform of the difference of the filtered image from the true image after the high-pass filtering. The cutoff frequency *k*_c _was chosen in each image so that most of the signal components are rejected in the calculation of *NERR*.

#### Beta metric (*β*)

As a measure to evaluate edge preservation and artifact formation, the beta metric (*β*) is defined by [9]:(13)

where Δ*I_n _*and *ΔI_f _*represent the edge images of the original image *I_n _*and the filtered image *I_f_*, respectively. The edge images are calculated by applying the 3 × 3 Laplacian operator followed by thresholding to reject false edges stemming from noise.  and  are the mean intensities of *ΔI_n _*and *ΔI_f_*, respectively. The beta metric represents the resemblance of the edge images derived from the original and filtered images. If the edge is well preserved during the denoising process, the beta metric will be close to unity.

##### SNR gain (*g*)

SNR is evaluated in five different uniform-intensity ROIs with the size of 10 × 10. The uniform region is selected manually in each image by visual inspection. SNR in the ROI is defined by the mean pixel intensity divided by the standard deviation of the pixel intensity in the ROI. The SNR gain is defined by:(14)

where *SNR_f _*and *SNR_n _*are the average SNRs in the filtered and original images, respectively.

#### Computation time

Computation time is evaluated on a personal computer with an Intel-Pentium^® ^Dual-Core CPU E6500 @ 2.93GHz processor. The filters have been implemented using MATLAB™ (The Mathworks, Inc., Natic, MA.) and the computation time is evaluated excluding the data readout time from the disk to the PC memory.

## Results

**Figure 4:**
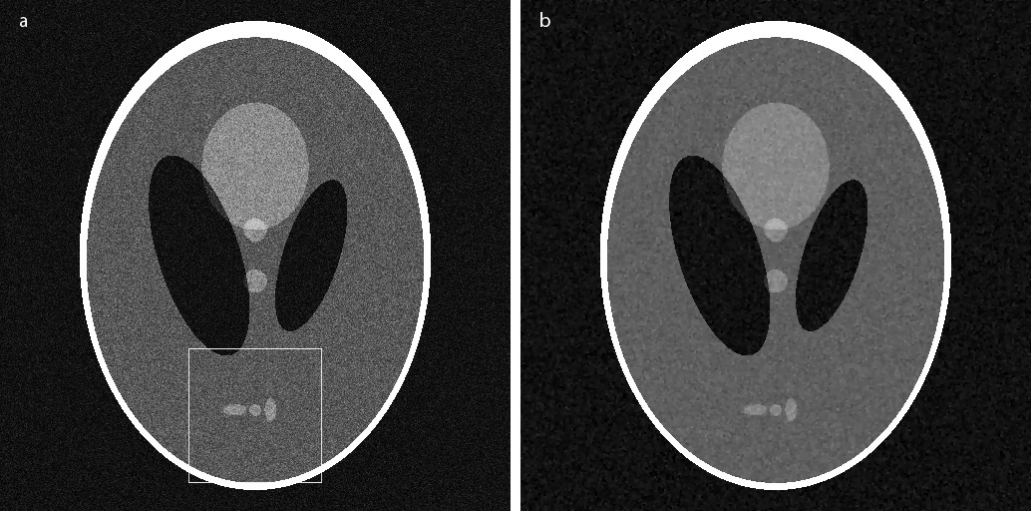
**Filtering of a synthetic image**. (a) The Shepp-Logan phantom corrupted by additive white Gaussian noise with standard deviation of 0.03. (b) The image filtered by the proposed method.

**Figure 5:**
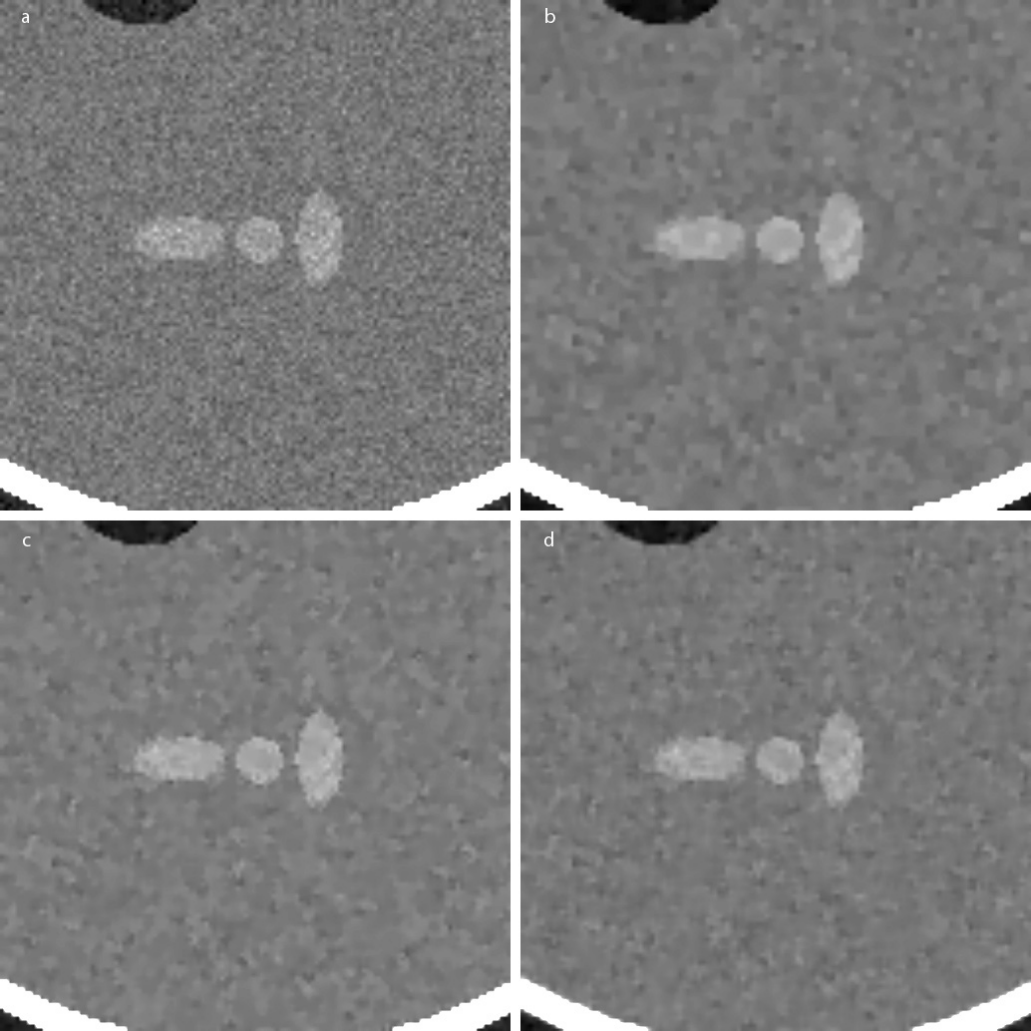
**Filtering of a synthetic image: comparison with ADFs**. (a) The original image with the noise added. (b-d) The images filtered by the proposed method, Perona-Malik ADF, and Weickert's ADF, respectively.

We first applied the proposed filter to denoising of synthetic images. As an original image, we used the Shepp-Logan head phantom image corrupted by the white Gaussian noise with the standard deviation of 0.03 as shown in Figure 4a. The image filtered by the proposed method is shown in Figure 4b. The number of iterations was four and the step size in each iteration was chosen to be 0.25 according to [14]. For the sake of comparison, we also filtered the original images with two types of ADFs, one Perona-Malik ADF and the other Weickert's ADF. In both cases we used the diffusion coefficient function defined in Eq. (2) and the median regularization method suggested in [18]. Figures 5a-d show zoomed ROIs images taken from the original image, and the images filtered by the proposed method, Perona-Malik ADF and Weickert ADF, respectively. Since the Shepp-Logan phantom image has simple structures in it, it is difficult to compare the filtering effects by visual inspection. Table 1 summarizes the filter performances of the three methods. As can be seen from Table 1, the proposed method shows the noise reduction and edge preserving performances similar to Perona-Malik ADF, but the computation time of the proposed method is far less than the other two methods.

**Table 1:** Performance of the proposed denoising filter in comparison with other ADFs

Images	Matrix size	NERR	NMSE	SNR gain	β	Computation time (s)
Shepp-Logan Phantom	512 × 512	0.979 (0.731) [0.927]	0.0885 (0.364) [0.196]	3.40 (2.43) [2.34]	0.987 (0.858) [0.964]	0.428 (1.050) [0.863]

Mouse Chest	2240 × 2240	0.942 (0.904) [0.903]	0.206 (0.393) [0.488]	2.10 (1.80) [1.74]	0.148 (0.271) [0.113]	8.072 (19.17) [15.45]

Micro-CT Sagittal	512 × 512			3.46 (2.92) [3.26]	0.809 (0.886) [0.986]	0.484 (1.26) [0.781]

Micro-CT Coronal	1024 × 1024			2.07 (2.05) [2.00]	0.894 (0.914) [0.902]	1.723 (3.989) [3.139]

Micro-CT Axial	512 × 512			2.19 (1.75) [1.36]	0.736 (0.803) [0.936]	0.470 (1.01) [0.949]

Human Chest	3072 × 3072	0.782 (0.778) [0.747]	0.376 (0.437) [0.470]	1.79 (1.65) [1.59]	0.163 (0.174) [0.166]	12.94 (34.69) [26.57]

( ) represents the results obtained with Weickert ADF.

[ ] represents the results obtained with Perona-Malik ADF.

**Figure 6:**
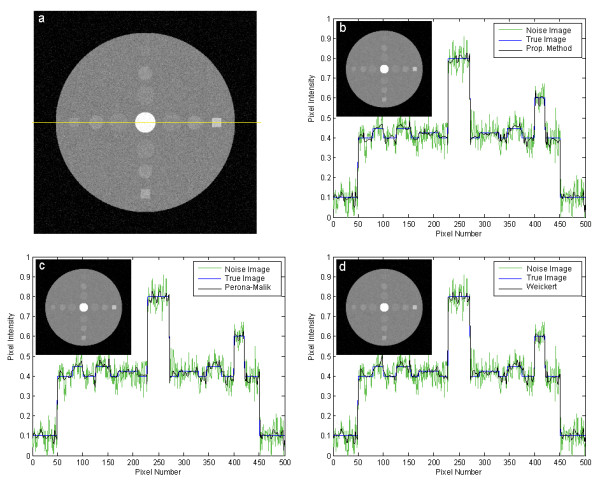
**Filtering of a synthetic image: comparison of edge preservation with ADFs**. (a) The original image with the noise added. (b-d) The images filtered by the proposed method, Perona-Malik ADF, and Weickert's ADF, respectively, along with profiles.

To demonstrate weak edge preservation performance, we have made a synthetic phantom shown in Figure 6a which has low contrast inclusions stained by heavy noise. We have applied the filters to remove the noise and we have shown the resulting images along with the cut viewing profiles in Figure 6b-d. The weakest contrast of the inclusions on the line is half the noise standard deviation. The proposed filter preserves the weak edges well as other ADFs do.

To test the proposed filter with real x-ray images, we first obtained 3D micro-CT images of a sacrificed rat using a lab-built micro-CT system. During the micro-CT scan, the micro-CT acquires 2D projection images at every angle which are to be used for 3D image reconstruction. Therefore, any of the 2D projection images can be thought to be a DR image. We reconstructed 3D tomographic images using the modified Feldkamp algorithm [19]. The micro-CT system consists of a micro-focus x-ray tube with a variable focal spot size of 5-50 μm (L8121-01, Hamamatsu, Japan), a CMOS flat-panel detector with a matrix size of 2240 × 2240 (C7942, Hamamatsu, Japan), a rotating subject holder for the cone-beam CT scan motion, and a data acquisition system. The pixel size of the flat-panel detector is 50 × 50 μm^2^. We performed the micro-CT scan with the tube voltage of 60kVp and the tube current of 340 μA which gave the nominal focal spot size of 50 μm. The number of projection views, which determines the angular spatial resolution of the micro-CT images, was 900. We reconstructed 3D micro-CT images with the matrix size of 512 × 512 × 512 with isotropic pixel size of 170 × 170 × 170 μm^3 ^or 1024 × 1024 × 1024 with isotropic pixel size of 85 × 85 × 85 μm^3^. Since the tube current of the micro-CT is two orders of magnitude smaller than those of medical CTs, micro-CT images usually suffer from high level noise in spite of long scan time.

**Figure 7:**
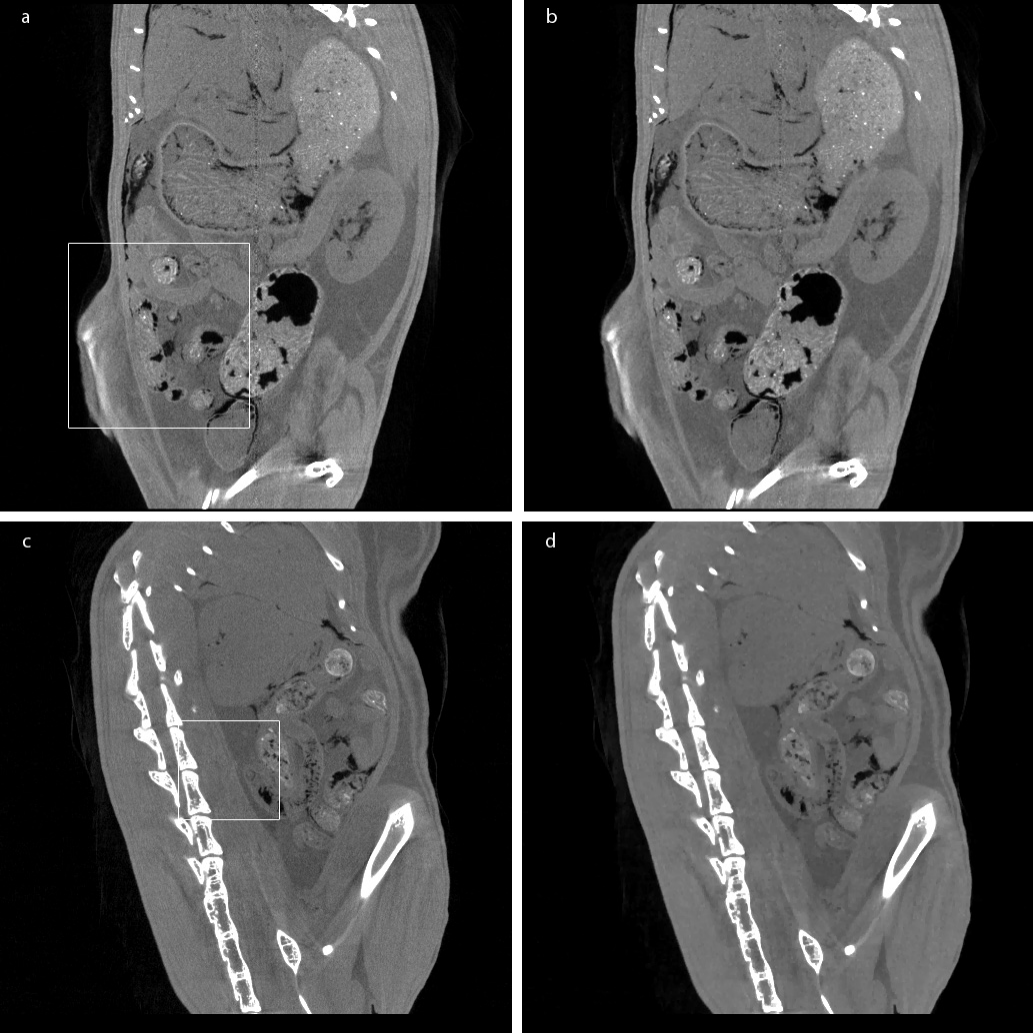
**Filtering of micro-CT images of a rat**. (a) A coronal image before filtering and (b) after filtering. (c) A sagittal image before filtering and (d) after filtering. The square boxes are the region of interest.

**Figure 8:**
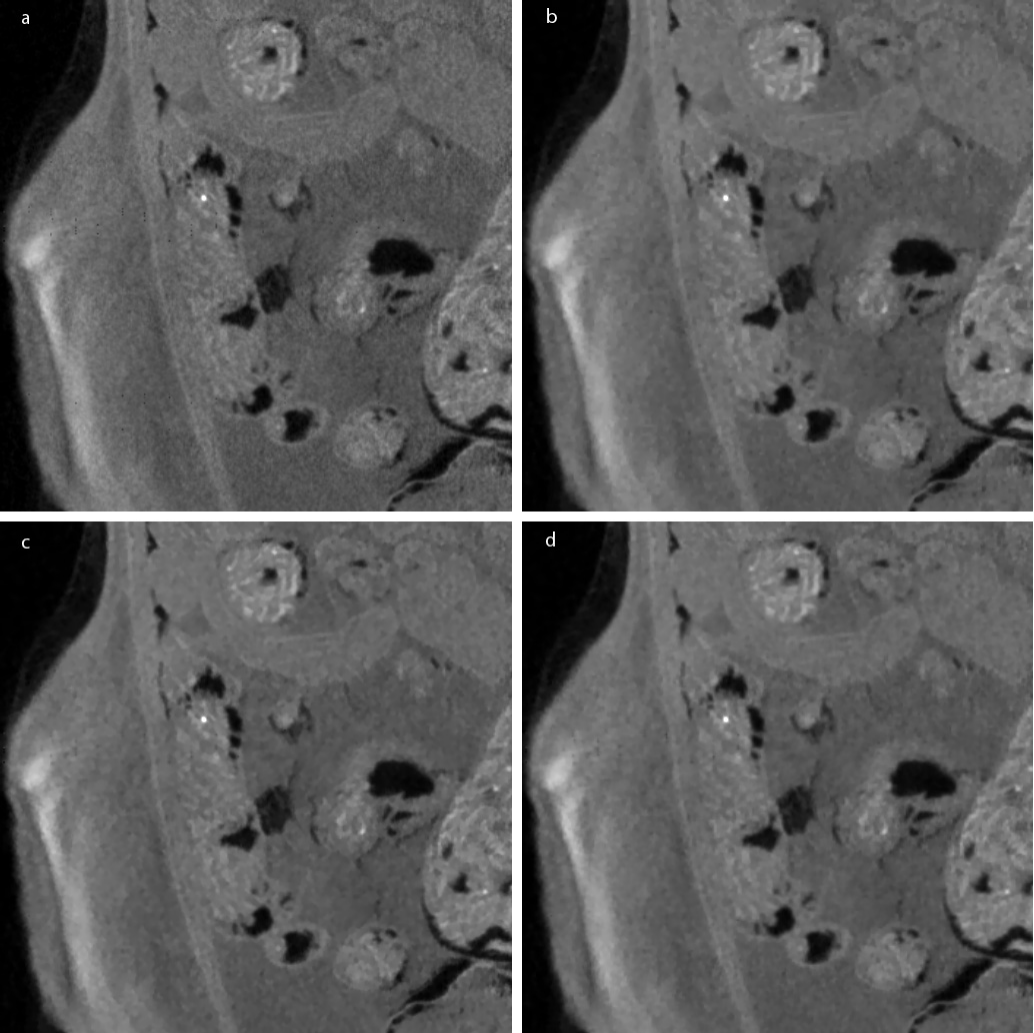
**Filtering of micro-CT images of a rat: zoomed coronal views**. (a) The original image. (b-d) The images filtered by the proposed method, Perona-Malik ADF, and Weickert's ADF, respectively.

**Figure 9:**
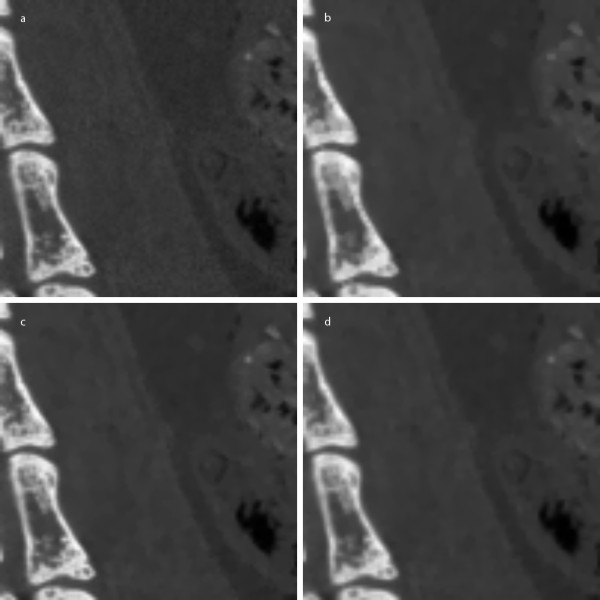
**Filtering of micro-CT images of a rat: zoomed sagittal views**. (a) The original image. (b-d) The images filtered by the proposed method, Perona-Malik ADF, and Weickert's ADF, respectively.

**Figure 10:**
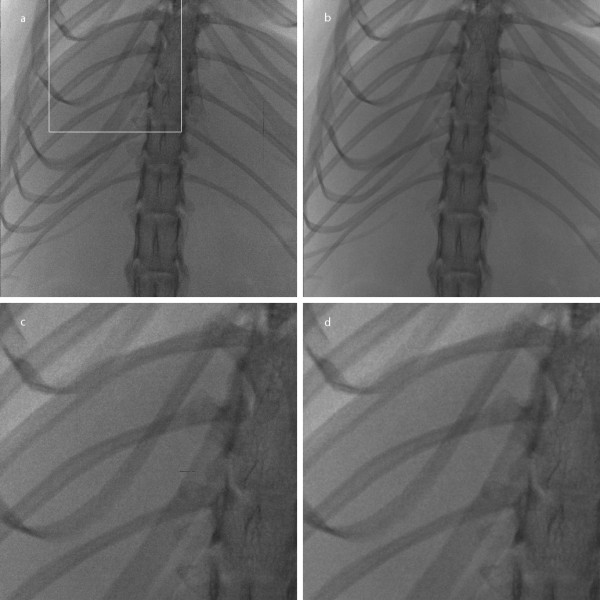
**Filtering of a DR image of a rat**. (a) A DR image of a rat before filtering and (b) after filtering. (c, d) The zoomed images of the ROI in (a) and (b), respectively.

Figure 7a shows an example of the micro-CT images of 1024 × 1024 × 1024 matrix size in coronal view and Figure 7c shows an example of the micro-CT images of 512 × 512 × 512 matrix size in sagittal view. Since the micro-CT images are in full 3D format, we can view the images in arbitrary direction. Figures 7b and 7d show the images filtered by the proposed method in four iterations. For better visualization of this images, magnified version of the ROIs are shown in Figure 8 and Figure 9. Here, we have also shown the images filtered by the two ADFs in four iterations and the same time step size of 0.25. As can be seen from Figure 8 and 9, the proposed filter well removes the noise while preserving small anatomic structures. Figure 10a shows a DR image of an adult rat taken with the tube voltage of 40kVp and the tube current of 67 μA. The exposure time was 1s. The image matrix size is 2240 × 2240 with the pixel size of 50 × 50 μm^2^. Figure 10c shows a magnified version of the ROI indicated by the white rectangle in Figure 10a. Figures 10b and 10d show the images filtered by the proposed method.

**Figure 11:**
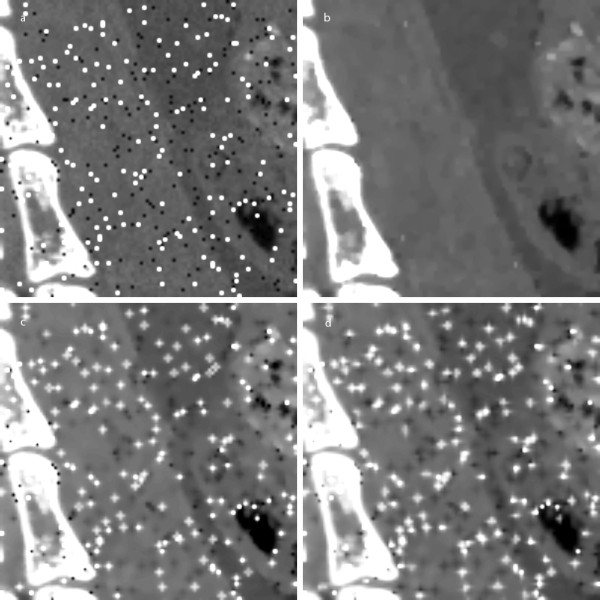
**Filtering of salt-and-pepper noise**. (a) A micro-CT image of a rat with the salt and pepper noises added. (b-d) The images filtered by the proposed method, Perona-Malik ADF, and Weickert's ADF, respectively.

Figure 11a shows the sagittal micro-CT image on which salt and pepper noises have been intentionally added. Salt and pepper noises may appear on DR images due to the defective detector elements. Figures 11b-d show the images filtered by the proposed filter and the two ADFs, respectively. Both the ADFs do not remove the salt and pepper noises completely whilst the proposed filter removes the noises very well.

Table 1 summarizes the filter performances in all the cases shown in the previous figures and also in extra cases of an axial-view micro-CT image of the rat and a DR image of a human chest. In the case of human chest imaging, we took the true image with much higher tube current of 2.2mA as compared to 0.4 mA in normal imaging. The tube voltage was 104 kVp. As can be noticed from Table 1, the overall performance of the proposed filter is similar to those of the two ADFs in terms of noise reduction and edge preservation. But, the computation time of the proposed filter is much shorter than the two ADFs.

## Discussion

The proposed filter uses the same form of PDE equation as the Perona-Malik ADF but with a different way of diffusion coefficient calculation. In the original Perona-Malik ADF, four different diffusion coefficients are employed to a given pixel in four orthogonal directions to allow different amount of diffusion in each direction according to the gradient strength. Owing to the four directional diffusion processes, the Perona-Malik ADF has better edge preservation than a linear diffusion filter. Even though the proposed filter uses only two diffusion coefficients in solving the PDE, it turns out that the proposed filter has similar performance to the Perona-Malik ADF. In Perona-Malik ADF, regularization must be incorporated into the diffusion coefficient calculation to stabilize the diffusion process avoiding the influence of high noises. Since the proposed filter is not based on the gradient-dependent diffusivity function with a global noise threshold, high impulsive noises do not affect the performance of the proposed filter as can be seen from the salt-and-pepper noise case in Figure 11. Even without any regularization in the proposed filter, we have not observed any unstable operations either in denoising the images we have used so far. Filtering without regularization has also allowed further reduction of computation time of the proposed filter. It is believed that the Weickert's ADF better removes the noise at the edge region, but it takes much longer computation time. Although fast algorithms to solve the Weickert's ADF have been proposed, those schemes suffer from bigger errors and artifacts [14]. There are also many other iterative non-linear denoising filters than ADFs, which are thought to outperform ADFs in many respects [20-22].

**Figure 12:**
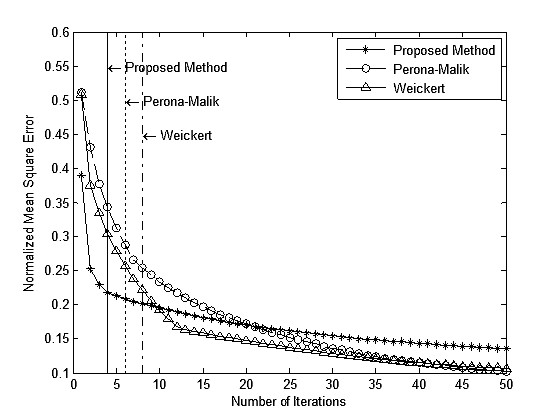
**Convergence of the filters**. Convergence behaviors of the proposed filter, Perona-Malik ADF and Weickert's ADF along with its respective optimal stopping points indicated by vertical lines.

Stopping iterations at the right time during the diffusion filtering process is an important issue. Weickert introduced a stopping criterion based on the variance of the filtered image relative to the variance of the original image [23]. Later, Mrázek proposed the so-called decorrelation criterion which minimizes the covariance of the image to the noise [24]. Bazan observed that the correlation between *I*(*t*) and *I*(0) decreases gradually from 1 to 0 as *t *→ ∝, and suggested that the iteration be stopped when the second derivative of correlation between *I*(*t*) and *I*(0) reaches a maximum that is very close to the correlation between the true and the filtered image [25]. We have found that Bazan's criterion agrees well with the behavior of the proposed filter. Figure 12 shows the NMSEs of the three filters calculated at every iteration step during the filtering of the rat image shown in Figure 10. It demonstrates that as the filtering process goes by, the proposed filter also converges to a steady state as the other methods. Even though the steady state error of the proposed filter is greater than the other methods, the proposed filter converges faster in the early stage of iterations. Based on the Bazan's criterion, the optimal number of iterations was four for this case which is smaller than those of the other filters. For other cases, the optimal number of iterations for the proposed filter ranges from 4 to 6 which always are smaller than those of the other filters. Until the iteration reaches the optimal point, the NMSE of the proposed filter is smaller than those of the other filters. We think faster convergence of the proposed filter is due to the use of local pixel topology in defining the diffusion coefficient, which makes the filter more robust to high intensity noises.

## Conclusions

We have experimentally confirmed that the proposed filter and the two ADFs have similar filter performance in terms of noise reduction and edge preservation. However, the proposed filter takes much shorter computation time than the two ADFs do. We expect the proposed filter can be greatly used to mitigate the noise effects in low-dose x-ray imaging.

## Competing interests

The authors declare that they have no competing interests.

## Authors' contributions

EM has devised the new filter design structure and implemented the proposed filter as well as the conventional ADFs using MATLAB™. He has also evaluated the filter performances using the synthetic and experimentally images. MH has guided EM in how to evaluate the filter performances. SY has obtained micro-CT images and DR images and he has also drafted and finalized the manuscript. All authors have read and approved the final manuscript.
